# Stability and genetic insights of the co-existence of *bla*_CTX-M-65_, *bla*_OXA-1_, and *mcr-1.1* harboring conjugative IncI2 plasmid isolated from a clinical extensively-drug resistant *Escherichia coli* ST744 in Shanghai

**DOI:** 10.3389/fpubh.2023.1216704

**Published:** 2023-08-23

**Authors:** Jun Feng, Huanyu Wu, Yuan Zhuang, Jiayuan Luo, Yong Chen, Yitong Wu, Jiayi Fei, Qi Shen, Zhengan Yuan, Min Chen

**Affiliations:** Shanghai Municipal Center for Disease Control and Prevention, Shanghai, China

**Keywords:** MCR-1, *Escherichia coli* STstrain-744 (ST744), colistin, stability, plasmid

## Abstract

**Background:**

Co-existence of colistin, β-lactam and carbapenem in multidrug-resistant *Enterobacteriaceae* isolates poses a serious threat to public health. In this study, we investigated and characterized the co-occurrence of *bla*_CTX-M-65_, *bla*_OXA-1_, and *mcr-1.1* strain isolated from a clinical extensively-drug-resistant *Escherichia coli* ST744 in Shanghai.

**Methods:**

Antimicrobial susceptibility test was carried out by agar dilution methods. Whole genome sequencing was conducted, and resistance genes, and sequence types of colistin in *E. coli* isolates were analyzed. Plasmid stability and amino acid mutations were assessed in *E. coli* isolates.

**Results:**

A colistin resistant *E. coli* ST744, named ECPX221, was identified out of 145 fecal samples collected. The strain carries a 60,168 IncI2 plasmid with the *mcr-1.1* gene. The strain also has *bla*_CTX-M-65_, *bla*_OXA-1_, *dfrA*14, *qnrS1*, *cmlA5*, *arr2*, *ampC*, *aph(4)-Ia*, *sul1*, and *aadA5* resistance genes. The plasmid pECPX221 was capable of conjugation with an efficiency of 2.6 × 10^−2^. Notably, 45% of the transconjugants were determined as *mcr-1.1*-harboring in the colistin-free environment after 60 generation of passage. No mutations occurred in *pmrB*, *mgrB*, and *phoPQ* gene in the *mcr-1.1*-harboring transconjugants. Bioinformatic analysis indicated pECPX221 shared highly similar backbone with the previously reported *mcr-1.1*-harboring pAH62-1, pMFDS1339.1, pSCZE4, and p2018-10-2CC. Furthermore, sequencing and phylogenetic analyses revealed a similarity between other MCR-1-homolog proteins, indicating that ECPX221 was colistin resistant.

**Conclusion:**

The stable transferable *mcr-1.1*-harboring plasmid found in the *E. coli* ST744 strain indicated the high risk to disseminate the extensively-drug-resistance phenotype among *Enterobacteriaceae*.

## Introduction

Multidrug resistance in *Escherichia coli* has become a concerning issue, displaying resistance to β-lactam antibiotics, particularly through the production of β-lactamases, including extended-spectrum β-lactamases (ESBLs) and carbapenemases (1). The increasing use of β-lactams and carbapenems over the past several decades has led to the increased use of colistin, which is now considered the last therapeutic option for treating infections caused by such organisms. However, the efficacy of colistin has been challenged by the emergence of plasmid-mediated mobile colistin resistance (*mcr-1*), which was found in *Enterobacteriaceae* in 2015 (2), and has since been disseminated in animals, meat products, humans (both fecal carriage and infections), and the environment in over 50 countries, covering six continents (3–5). In China, colistin has been adopted for treating carbapenem-resistant *Enterobacteriaceae* (CRE) infections since 2018 (6), thereby increasing the potential risk of the dissemination of *mcr-1* (7). To date, *mcr-1* positive plasmids have also been found in multidrug-resistant (MDR) and extensively drug-resistant (XDR) *Enterobacteriaceae* isolates carrying plasmid-borne carbapenemase and ESBL genes (8), which could generate resistance to multiple drugs and contribute to the spread of MDR bacteria in human populations (9).

Previous studies have reported *mcr-1*-harboring plasmids found in different Inc. types, while IncI2, IncHI2, and IncX4 appear to be the most common carriers of *mcr-1* (3, 10–12). The mobility of *mcr-1* is difficult to control because *mcr-1*-harboring plasmids often carry other antimicrobial resistance (AMR) genes, including those encoding resistance to β-lactams, fluoroquinolones, and tetracyclines (8, 13, 14). For example, Wang et al. reported *E. coli* strain QE11-421, which was isolated from a sputum sample of a 90-year-old male patient receiving treatment in the ICU, was an *mcr-1*-positive colistin-resistant isolate that co-harbored the *bla*_KPC-2_ gene conferring carbapenem resistance (15). Zhang et al. (16) reported the coexistence of the *bla*_NDM-5_, *bla*_CTX-M-65_, *bla*_OXA-10_, *bla*_TEM-1_ and *mcr-1.1* genes detected in *E. coli* 20IR1127 strain belonging to ST156 lineage isolated from children in China. Lu reported three *E. coli* ST6775 coharboring *tet*(X4), *mcr-1*, and *bla*_NDM-5_ isolates from pigeons (17)_._ In this study, we investigated the prevalence of colistin-resistant *E. coli* isolates from fecal samples in Shanghai and characterized the plasmid stability and persistence that contribute to colistin resistance in *E. coli* isolates.

## Materials and methods

### Sample collection

Fresh fecal samples were collected from patients experiencing acute diarrhea within 3 days of symptom onset, and who had not received any antibiotics treatment from September 2021 to January 2022 from surveillance hospitals in Shanghai. The samples were collected using five sterile cotton swabs from multiple sites and placed into a 50 mL screw-cap-sealed centrifuge tube with C–B transport medium. For patients and infants who experienced difficulty in defecating, rectal swabs were used to collect the samples. The rectal cotton swab was soaked in normal saline and inserted 4–5 cm deep into the anus (2–3 cm for children), and gently rotated. At least two swabs were collected from the same patient and placed in C–B transport medium and 3 mL of preservation solution (containing 5% bovine serum cell maintenance solution). The samples were stored at 4°C and sent to the laboratory within 48 h after collection.

### Bacterial strains, identification and *mcr-1* gene screening

Each fecal sample was placed in sterile plastic bags containing 225 mL of Mueller–Hinton broth and incubated overnight at 37°C. The samples were then seeded on Nutrient broth plates (COMAGAL Microbial Technology, Shanghai) with 2 μg/mL colistin and incubated for 24 h at 37°C. To identify the isolate, a positive colony was selected by amplifying the *mcr-1* gene via real-time PCR (RT-PCR). The strains were then identified by MALDI-TOF mass spectrometry using the VITEK MS system (BioMérieux Shanghai Co. Limited). Basic clinical data, including gender, age, and date of isolation, were collected for patients from whom the *mcr-1*-harboring strains were isolated. The *mcr-1* gene screening was performed as following: briefly, the genomic DNA from each of the strains was extracted by boiling and freeze-thawing processes, and the resulting supernatant was used as the template. The specific primer used in this study was: *mcr-1*-RT-F: 5′-CGCGATGCTACTGATCACCA-3′, *mcr-1*-RT-R: 5′-GGTCGTATCATAGACCGTGCC-3′, and the *mcr-1*-probe: VIC-5′-TTATCATCGTATCGCTATGTGCTA-3′-MGB.

### Antimicrobial susceptibility testing

A total of 30 antimicrobial agents (Shanghai Fosun Biological Technology Co., Ltd.) were used for antimicrobial susceptibility testing via broth microdilution method, including ampicillin (AMP), ampicillin/sulbactam 2:1 ratio (AMS), tetracycline (TET), chloramphenicol (CHL), trimethoprim/sulfamethoxazole (SXT), cefazolin (CFZ), cefotaxime (CTX), ceftazidime (CAZ), cefoxitin (CFX), gentamicin (GEN), imipenem (IMP), nalidixic acid (NAL), azithromycin (AZI), tigecycline (TIG), ciprofloxacin (CIP), amoxicillin/clavulanic acid (AMC), cefotaxime/clavulanic acid (CTC), ceftazidime/clavulanic acid (CAC), colistin (CT), aztreonam (ATM), cefuroxime (CXM), amikacin (AMI), cefepime (CPM), meropenem (MEM), levofloxacin (LEV), ertapenem (ETP), ceftazidime/avibactam (CZA), streptomycin (STR), and norfloxacin (NOR). The double-disc synergy test and a modified carbapenem inactivation method were used to confirm the production of ESBL and carbapenemase, respectively, according to Clinical and Laboratory Standards Institute (CLSI) guidelines. *E. coli* ATCC^®^25922^™^ strain was used as quality control. The European Committee on Antimicrobial Susceptibility Testing (EUCAST) breakpoints defines colistin resistance as 2 μg/mL for *Enterobacteriaceae* (18).

### Conjugation assay

To investigate whether the *mcr-1* gene was present on a transferable plasmid, we performed a filter mating assay using rifamycin-resistant *E. coli* C600 as the recipient strain. Both the original isolates and recipient *E. coli* C600 were grown overnight in LB broth and adjusted to a 0.5 McFarland standard. The donor bacteria were mixed with recipient *E. coli* C600 at a ratio of 1:3 to 5 mL of fresh LB broth and then incubated at 37°C overnight. Transconjugants were selected on LB plates supplemented with rifampicin (40 μg/mL) and colistin (2 μg/mL). Putative transconjugants were confirmed using PCR and antimicrobial susceptibility tests. The mobilization efficiency were calculated by dividing the number of transconjugant colonies by the number of donor colonies (19).

### Plasmid stability assay

The plasmid stability assay was conducted to determine the stability of the *mcr-1*-harboring plasmids in the absence of antibiotic selective pressure. The strains were grown in LB broth containing colistin (2 μg/mL) and then transferred to a fresh LB broth without antibiotics. The cultures were periodically passaged for 60 days, with serial passages performed every 24 h, resulting in approximately 600 generations of bacterial growth. Cultures from passages 2, 4, 6, 8, 10, 20, 30, 40, 50, and 60 were diluted and plated onto LB plates containing colistin and LB plates without antibiotics. The frequency of stable plasmids was calculated as the number of colonies grown on the LB plate containing colistin and the antibiotic-free LB plate, divided by the total number of colonies on both plates, multiplied by 100%. The distribution of all *mcr-1*-harboring plasmids from different antimicrobial environments and passages was monitored by RT-PCR and Sanger sequencing to identify any changes in the plasmid composition over time.

### Genetic mutation in colistin-resistant isolates

To assess the genetic mutation in the colistin-resistant *E. coli* isolates, the colistin resistance genes including *mgrB*, *pmrAB*, *phoPQ* were amplified by real-time quantitative PCR as previously described (20, 21). Mutations that occurred in colistin-resistant *E. coli* isolates were determined by comparing to their corresponding parental reference genomes.

### Whole genome sequencing

The genomic DNA was extracted and sequencing libraries were generated using the TruSeq DNA Sample Preparation Kit (Illumina, USA). The genome sequencing was then performed with standard protocol and were sequenced with 150-bp paired-end strategy by using the Illumina Novaseq 6,000 (Sangon Biotech Company, Shanghai, China). Data assembly was carried out after adapter contamination removal and data filtering by using AdapterRemoval and SOAPec. Scaffold and contig construction were performed using SPAdes (version 3.12.0) and A5-miseq, respectively, with integration of all assembled results to obtain a complete sequence.

Subsequently, the VFDB (Virulence Factors of Pathogenic Bacteria)^1^ and CARD (The Comprehensive Antibiotic Resistance)^2^ database were used to retrieve the pathogenicity genes and antibiotic resistance genes, respectively. The *mcr-1*-carrying contigs generated by Illumina sequencing were examined for Inc. types by PlasmidFinder version 2.1.^3^ The set of close typing sequences was determined by PubMLST^4^ and then compared with the typing sequences of *Escherichia* spp. The linear comparison of complete plasmid sequences was created by EasyFig version 2.2.2.^5^ The plasmid construction map was generated by SnapGene 6.1.2 software (Insightful Science, United States). The genome-wide similarities was generated by FastANI version 1.33.^6^ Sequences were deposited to NCBI website under the Bioproject PRJNA929103.

### Phylogenetic analysis

The MCR-1 and MCR-1-like proteins’ homologous sequences were extracted from NCBI through BLASTp search (https://blast.ncbi.nlm.nih.gov/Blast.cgi, accessed on 20 June 2023), with MCR-1 protein of ECPX221 in this study obtained from the sequencing data. Aligned sequences of MCR-1 obtained from ClustalW version 2.0^7^ were used to construct a phylogenetic tree through the Maximum Likelihood Method of MEGA X (Mega Limited, Auckland, New Zealand). To confirm the results, 1,000 bootstrap repetitions were used.

### Ethical considerations

This study was reviewed and approved by the ethical committee of the Shanghai Municipal Centre for Disease Control and Prevention.

## Results

### Antimicrobial susceptibility profile of *mcr-1*-harboring *Escherichia coli* isolates

Out of 145 fecal samples collected between September 2021 and January 2022, only one *E. coli* isolate, named ECPX221, was found to harbor the *mcr-1.1* gene. Antimicrobial resistance testing was performed on this *mcr-1.1*-positive strain, and it was found to exhibit colistin resistance at 2 μg/mL (as shown in Table 1). Furthermore, ECPX221 was identified as an extended-spectrum β-lactamase producer, and was found to be resistant to CIP, AMP, AMS, CFZ, CTX, CXM, SXT, NAL, CHL, TET, ATM, LEV, and NOR. However, it was found to be susceptible to 15 other common antibiotics, including CFX, CPM, CZA, IMP, ETP, TIG, and others (as shown in Table 1). Apart from *mcr-1.1*, ECPX221 was also found to carry resistance genes for *bla*_CTX-M-65_, *bla*_OXA-1_, *dfrA*14, *qnrS*1, *cmlA*5, *arr*2, *ampC*, *aph(4)-Ia*, *sul*1, and *aadA*5.

**Table 1:** Information for the *mcr-1.1*-harboring *Escherichia coli* strain ECPX221 identified in this study and the transconjugant isolate ECPX221-T. The wild type EC C600 was used as control.

Antibiotic	ECPX221		ECPX221-T		EC C600	
	MIC	Result	MIC	Result	MIC	Result
CIP	4	R	8	R	≤0.015	S
AMP	>64	R	>64	R	4	S
AMS	32/16	R	16/8	I	4/2	S
CT	2	I	4	R	0.25	S
CFZ	>32	R	>32	R	2	S
CTX	16	R	16	R	≤0.25	S
CAZ/C	0.5/4	–	≤0.25/4	–	≤0.25/4	–
CTX/C	1/4	–	≤0.125/4	–	≤0.125/4	–
CFX	8	S	2	S	2	S
CPM	2	S	≤1	S	≤1	S
CXM	>32	R	>32	R	≤0.5	S
CZA	≤0.25/4	S	≤0.25/4	S	≤0.25/4	S
IMP	≤0.25	S	≤0.25	S	≤0.25	S
CAZ	1	S	2	S	0.5	S
AZI	8	–	4	–	≤2	–
ETP	≤0.25	S	≤0.25	S	≤0.25	S
SXT	>8/152	R	>8/152	R	>8/152	R
NAL	>64	R	>64	R	>64	R
CHL	>64	R	32	R	4	S
GEN	2	S	32	R	≤1	S
TET	>32	R	>32	R	≤1	S
TIG	0.5	S	≤0.25	S	≤0.25	S
AMK	4	S	4	S	≤2	S
ATM	16	R	16	R	≤2	S
LEV	>4	R	>4	R	≤0.125	S
MEM	≤0.125	S	≤0.125	S	≤0.125	S
STR	>32	–	>32	–	8	–
NOR	16	R	32	R	≤0.125	S

Ampicillin (AMP), ampicillin/sulbactam 2:1 ratio (AMS), tetracycline (TET), chloramphenicol (CHL), trimethoprim/sulfamethoxazole (SXT), cefazolin (CFZ), cefotaxime (CTX), ceftazidime (CAZ), cefoxitin (CFX), gentamicin (GEN), imipenem (IMP), nalidixic acid (NAL), azithromycin (AZI), tigecycline (TIG), ciprofloxacin (CIP), amoxicillin/clavulanic acid (AMC), cefotaxime/clavulanic acid (CTC), ceftazidime/clavulanic acid (CAC), colistin (CT), aztreonam (ATM), cefuroxime (CXM), amikacin (AMI), cefepime (CPM), meropenem (MEM), levofloxacin (LEV), ertapenem (ETP), ceftazidime/avibactam (CZA), streptomycin (STR), and norfloxacin (NOR).

### Transmissibility of *mcr-1.1* via conjugation

The result indicated that the *mcr-1.1*-harboring plasmid was capable of successful transfer from the donor strain to the recipient strain (*E. coli* C600). The conjugation of ECPX221 to *E. coli* C600 via horizontal transfer was achieved with an average efficiency of 2.6 × 10^−2^. The transconjugant ECPX221-T, which was confirmed to harbor *mcr-1.1* gene, exhibited a MIC value of 4 μg/mL to colistin, which represented a significant increase when compared to the wild type *E. coli* C600 (0.25 μg/mL). Therefore, it was speculated that the transconjugants acquired the colistin resistance gene from the donor strain.

### Plasmid stability and genetic mutations

**Figure 1:**
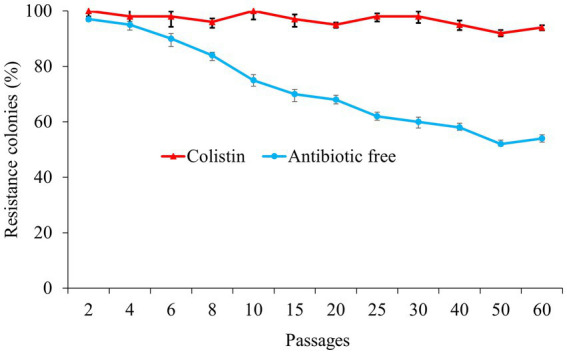
The resistance colonies that harboring *mcr-1.1* gene in the colistin (red) and antibiotic free environment (blue).

In order to assess the plasmid stability, we analyzed the dynamics of the pECPX221 plasmid by passaging the ECPX221-T strain carrying the plasmid with colistin for 60 days. Next day, 97% of the transconjugants (29 positive colonies out of 30 colonies) were detected as *mcr-1.1* positive in an antibiotic-free environment, while all transconjugants were positive in the colistin environment. On day 30, only 60% of the transconjugants (15 positive colonies out of 25 colonies) were detected as *mcr-1.1*-carrying isolates in the antibiotic-free environment, and this result decreased further to 45% on day 60, while the positive rate remained at 94% in the plate with colistin (Figure 1). To assess genetic mutations related to colistin resistance in the *mcr-1*-harboring plasmid in *E. coli*, we examined key genes such as *mgr*B, *pmr*AB, and *pho*PQ in the *mcr-1.1*-harboring and non-harboring transconjugants. However, our results showed that none of the aforementioned genes were mutated in the *mcr-1.1*-harboring transconjugants.

### Molecular features of *mcr-1.1* harboring strain

**Figure 2:**
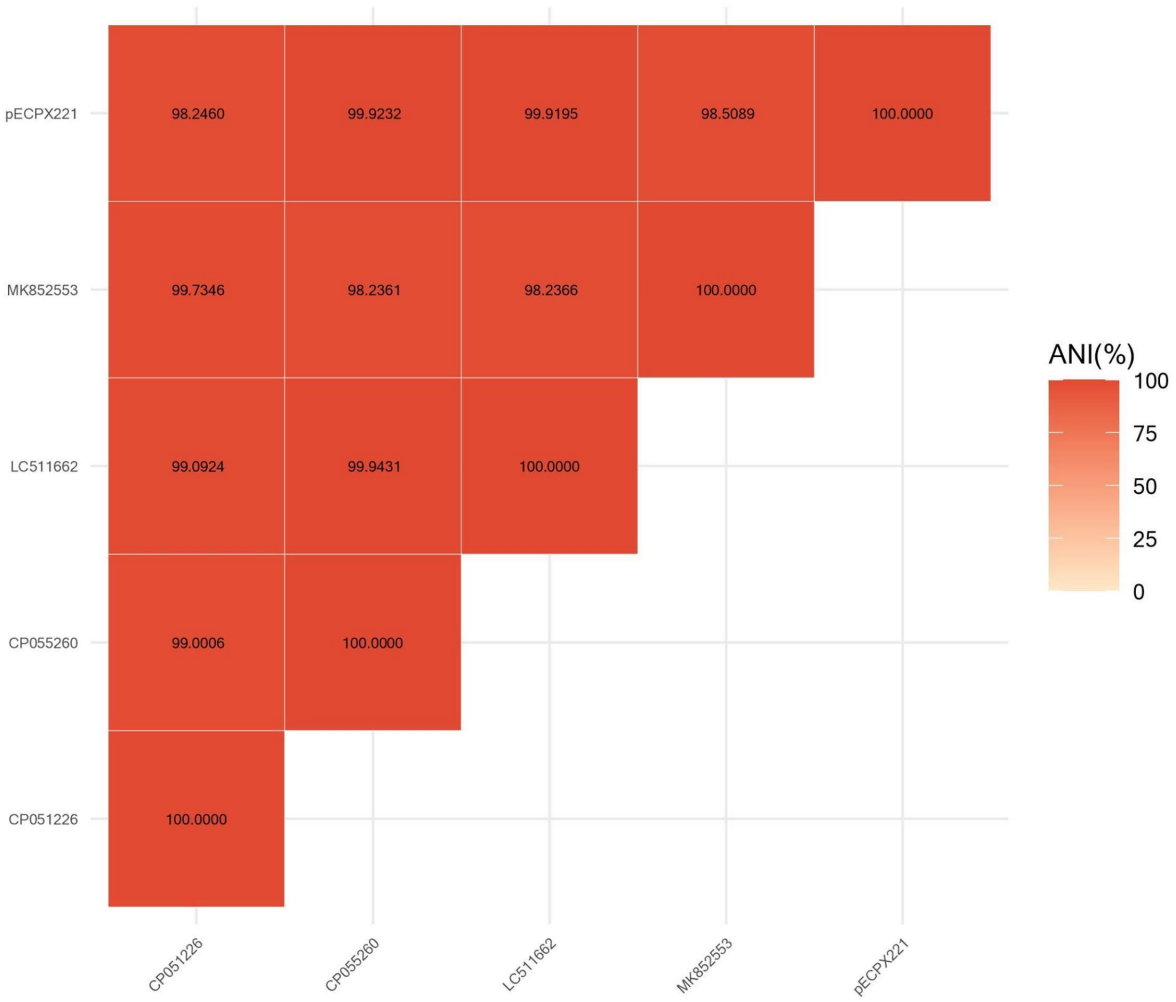
Comparison of pECPX221 and other *mcr-1*-harboring plasmids including pAH62-1, pMFDS1339.1, pSCZE4, and p2018-10-2CC by FastANI software.

**Figure 3:**
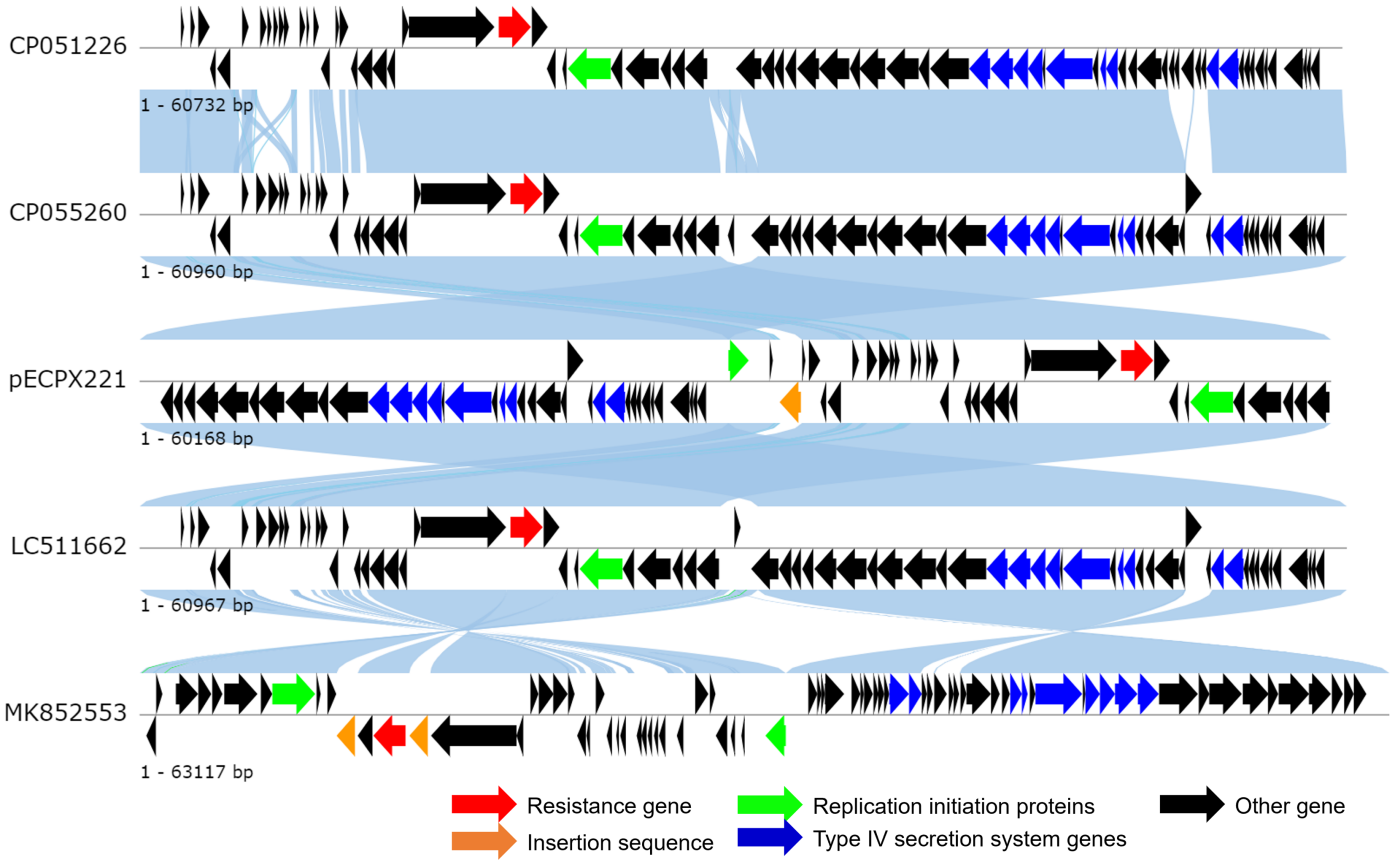
Linear comparison of complete plasmid sequences of plasmid *Escherichia coli* pECPX221 (this study), pAH62-1 from *E. coli* AH62 (GenBank Accession No. CP055260), pMFDS1339.1 from *E. coli* MFDS1339 (GenBank Accession No. MK852553), pSCZE4 from *E. coli* SCZE5 (GenBank Accession No. CP051226), and p2018-10-2CC from *E. coli* 2018–10-2CC (GenBank Accession No. LC511662).

**Figure 4:**
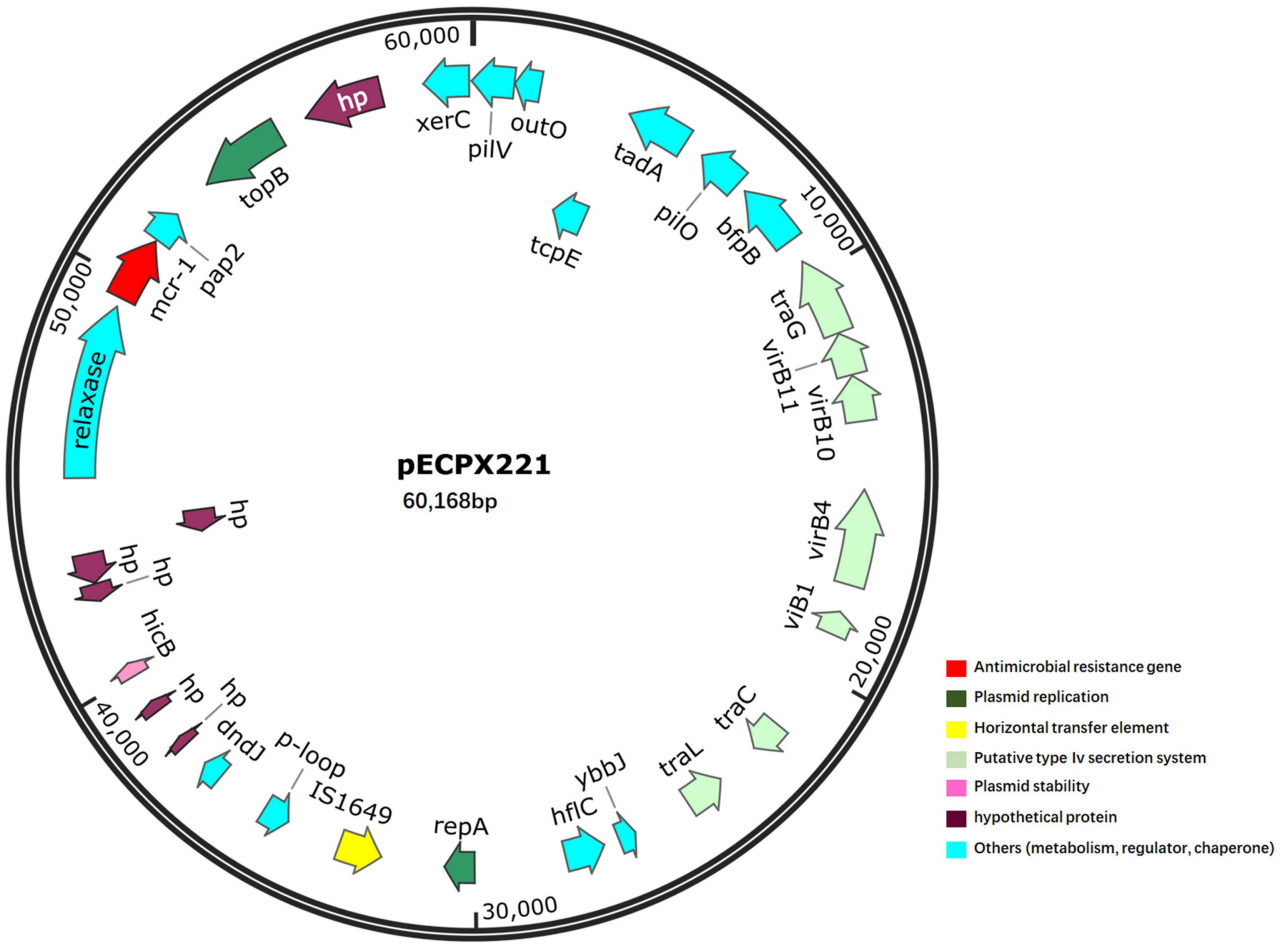
Map of *mcr-1*-harbouring plasmid pECPX221. The *mcr-1* gene is marked in red.

We sequenced the genomes of the EXPX221 and the sequence data showed that it belonging to the replicon types IncI2. BLASTn analysis showed that the backbone of the plasmid pECPX221 (GenBank Accession No. GCA_028527545) was strikingly similar with (the query cover of 100% and the identities 99%) other previously sequenced *mcr-1.1*-harboring IncI2 plasmids, such as pAH62-1 from *E. coli* AH62 (GenBank Accession No. CP055260), pMFDS1339.1 from *E. coli* MFDS1339 (GenBank Accession No. MK852553), pSCZE4 from *E. coli* SCZE5 (GenBank Accession No. CP051226), and p2018-10-2CC from *E. coli* 2018–10-2CC (GenBank Accession No. LC511662). In all, the ANI heatmap showed that these IncI2 plasmids bearing *mcr-1.1* showed very high architectural conservation (Figure 2). Furthermore, the BLAST comparison of pECPX221, pAH62-1, pMFDS1339.1, pSCZE4 and p2018-10-2CC revealed that their *mcr-1.1* insertion sites differed (Figure 3). An approximately 2.5 kb *mcr-1.1-pap2* element was identified in the above-mentioned plasmids. In addition, an integrase core domain protein as IS481-like element ISEc19 family transposase (WP_010723086) was identified in pECPX221, but only found in pMFDS1339.1. The putative conjugal transfer components of pECPX221 were also detected by using oriTfinder. The *vir* gene family encoding VirB1 to VirB11 were identified as T4SS belonging to Type IV secretion system was predicted on pECPX221 (Figure 4). The relaxase in pECPX221 from 45,033 to 49,346 nt was found to be 99% identity with the relaxase (WP_124777228.1) that obtained from *E. coli*. This evidence confirms that pECPX221 is a conjugative plasmid.

### Phylogeny analysis

**Figure 5:**
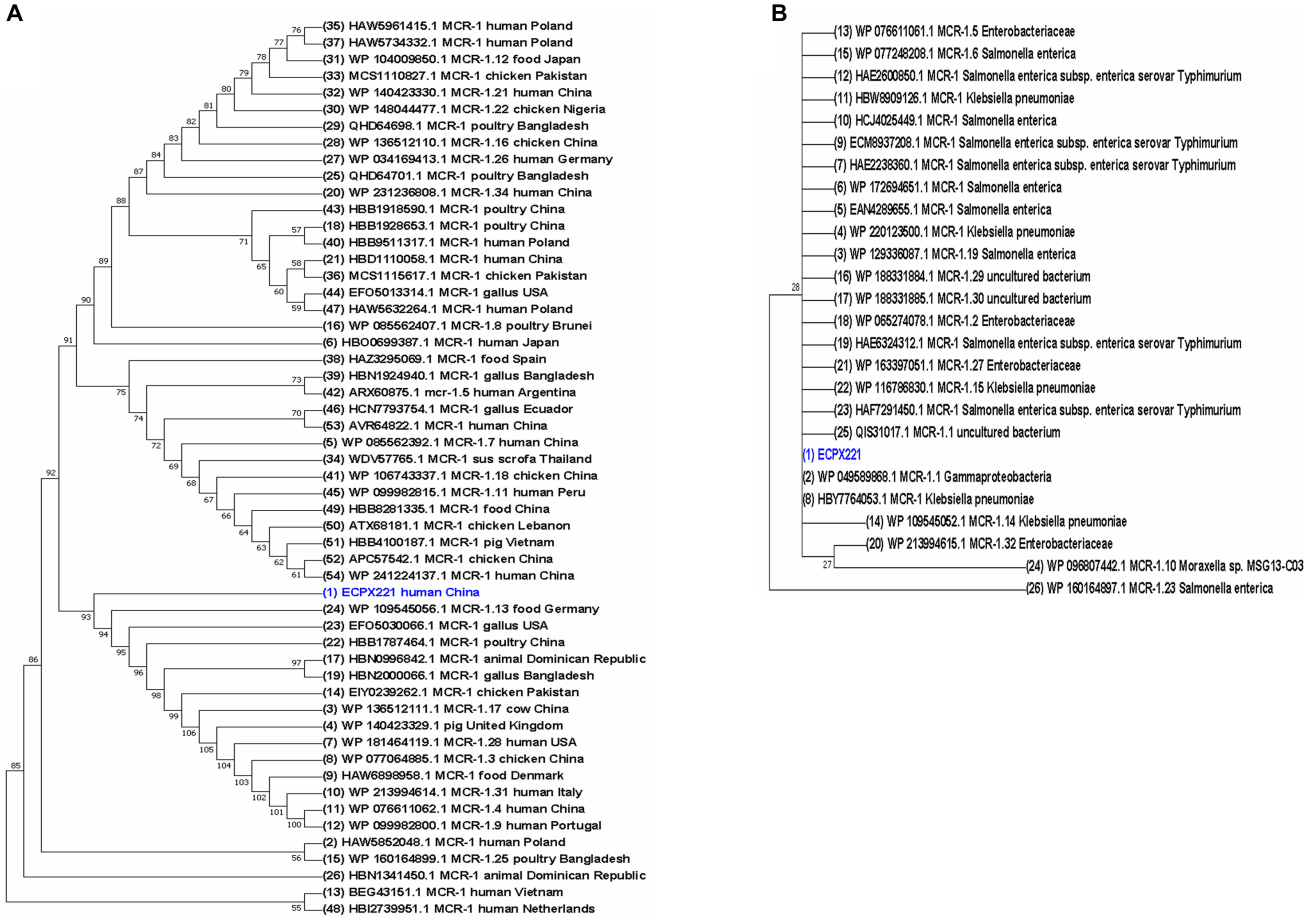
Phylogenetic study of MCR-1 and MCR-1-like proteins reveals ancestral origins and diversification. Using amino acid sequences from MCR-1 protein of ECPX221 in this study, the BLAST search tool (https://blast.ncbi.nlm.nih.gov/Blast.cgi, Accessed on 20 June 2023) was used to retrieve related sequences of MCR-1 and MCR-1-like proteins from the NCBI database. MCR-1 and MCR-1-like proteins of *E. coli*, *Salmonella*, and strains containing LptA and others were among the sequences categorized. Using aligned MCR-1 sequences from CLUSTALW, the maximum likelihood method of MEGA X was used to create a phylogenetic tree. **(A)** Phylogeny of 54 MCR-1 proteins of *E. coli* origin retrieved from NCBI database including sequenced MCR-1 gene of ECPX221 from this study. **(B)** Phylogeny of 26 MCR-1 proteins retrieved from NCBI database except *E. coli* strains.

A total of 54 MCR-1 proteins originated from *E. coli* (Supplementary File S1), and 26 proteins of MCR-1 gene obtained from bacteria other than *E. coli* (Supplementary File S2) were categorized for further analysis. In the case of protein acquisition from the NCBI database, above 50% of query coverage was set as the screening point. These two sets of proteins (Supplementary Files S1, S2), including ECPX221 strain harboring MCR-1 in this study, were used for phylogenetic analysis (Figure 5). The sequenced MCR-1 of ECPX221 in this study clearly showed its genomic confirmation as *mcr-1* genes by highly aligning with *mcr-1* genes of *E. coli* as well as other bacteria origins. In addition, the phylogenetic tree showed that ECPX221 strain in this study was mostly of Asian origin and that they were closely related.

## Discussion

Colistin resistance has become a serious issue in food animals such as pigs and chickens, as it has been frequently used since the 1950s (22, 23). Livestock and poultry are known to be the main reservoir for colistin resistance, and the discovery of the stable plasmid-mediated *mcr-1* gene in *E. coli* has helped us understand the potential transmission of colistin resistance between animals and humans (2). In this study, we have identified the co-occurrence of ESBL genes (*bla*_CTX-M-65_ and *bla*_OXA-1_) and *mcr-1.1*-producing *E. coli* ST744 isolates from clinical fecal samples in Shanghai. The plasmid pECPX221 contains four typical conjugal modules: a T4CP gene, an *ori*T-like region for transfer origin, a relaxase gene, and a gene cluster for the bacterial T4SS apparatus. Additionally, pECPX221 contains the *mcr-1.1-pap2* cassette, which has been shown to be capable of horizontally transferring into various plasmid replicon types (24).

The prevalence of coexistence of *mcr-1,* ESBL genes carrying *E. coli* was found to be only 0.7% (1/145) in clinical fecal samples, which is similar as that one *E. coli* strain harboring *mcr-1* and *bla*_NDM-5_ reported in companion animals that was collected from six different cities including Harbin, Yangzhou, Chongqing, Wuhan, Chengdu and Guangzhou (0.8%, 1/129) (25). Also, it was not that high when comparing with that has been reported in other regions in China such as in Shandong (3.5%) (26), Shanghai (3.9%) (27), Henan (7.7%) (28) and some other countries like in Pakistan (23.2%) (29), Lebanon (18%) (30), Japan (4.84%) (31) and Bolivia (38.3%) (32), but a little higher than that children patients with diarrhoea in Shanghai (0.28%) (33). This low prevalence could be attributed to the strict usage of colistin in Shanghai. According to the Shanghai Catalogue of Graded Management of Clinical Application of Antibacterial Drugs (2021 version) (34), colistin is only used for extensively resistant gram-negative infections such as *Pseudomonas aeruginosa*, *Acinetobacter baumannii*, and *Enterobacteriaceae*.

*E. coli* is the first and most prevalent species carrying the *mcr-1* gene in *Enterobacteriaceae* and can be isolated from raw meat and fecal samples of animals and humans in China (2, 35). The co-existence of ESBL genes such as *bla*_CTX-M-65_ and *mcr-1*-harboring *E. coli* have been reported globally, particularly in animal source *E. coli* isolates in northern China (36, 37). In eastern China, Zhejiang Province identified the co-occurrence of *bla*_NDM-5_, *bla*_CTX-M-65_, *bla*_OXA-10_, *bla*_TEM-1_, and *mcr-1.1* genes isolated from human *E. coli* ST156 in 2022 (16). Jiangsu Province reported an ESBL, carbapenemase- and *mcr-1*-producing *E. coli* ST648 strain isolated from a urine sample, which was found to have three transferable resistance plasmids (38). Apart from *E. coli*, one isolate named CFSA664 was found to co-harbor the *mcr-1* gene and *bla*_CTX-M-65_ in *Salmonella enterica serotype* Indiana from retail chickens in Jiangsu (39). However, the co-existence of *bla*_CTX-M-65_ and *mcr-1* was found in *E. coli* ST117 isolated from a veterinary hospital in Shanghai (40) and EC1CT136A isolated from broiler farms in Ecuador (41). Our study identified, for the first time, the co-existence of *bla*_CTX-M-65_, *bla*_OXA-1_, and *mcr-1.1* from a human fecal isolate in *E. coli* ST744 in Shanghai. This finding indicates a high risk of disseminating this extensively drug-resistant *E. coli*, which poses a threat to public health.

WGS data indicated the presence of various determinants of antibiotic resistance, suggesting that carbapenem and colistin co-resistant strains may be selected with the use of any antibiotics. Colistin resistance typically arises from selective pressure resulting from the use of polymyxins (2). In China, colistin was widely used as a growth promoter in animal production until 2017, when it was banned due to the identification of plasmid-mediated colistin-resistant isolates in the country (42, 43). Furthermore, colistin therapy in humans was introduced in February 2017 (7). As a result, it is essential to note that the prevalence of colistin-resistant isolates may be underestimated, as colistin susceptibility is not routinely tested in clinical samples from outpatients.

Plasmid horizontal transfer of *mcr-1* gene was widely reported in human, food, animals, and the environment in a lot of countries and regions worldwide (44). In this study, the plasmid replicon type of IncI2, the major type of plasmids spreading globally that promote *E. coli* resistance, was identified. The IS*Apl*1, which is consistently associated with the *mcr-1* gene and its related cassette that can be inserted into a variety of genetic loci in different plasmids, was missing in our study, this was similar to the previously reports on the absence of IS*Apl*1 in *E. coli* strain and other *Enterobacteriaceae* such as *E. fergusonii* (45). The diversity of plasmids carrying the *mcr-1* gene has been shown to increase with the use of colistin in clinical settings, suggesting that colistin administration could promote the dissemination of diverse resistance plasmids among *E. coli* isolates (46). The result of plasmid stability assay revealed that pECPX221 remained stable in the recipient bacterial strain, which was consistent with other *E. coli* strains (47, 48).

The phylogenetic analysis indicated that the sequenced *mcr-1* genes of *E. coli* are homologous to previously reported *mcr-1* genes from *E. coli* and other bacteria origins. The sequence of the *mcr-1* identified in our study is identical to that of the *mcr-1* previously identified from *E. coli* (GenBank Accession No: A0A0R6L508). The phylogenetic tree demonstrates a close relation among some studied *mcr-1* strains, and an evolutionary relationship to other *mcr-1* genes like strain WP109545056, EF05030066, and HBB1787464. Furthermore, the phylogenetic tree revealed that the *mcr-1*-positive ECPX221 in this study is related to many MCR-1 variants. For example, it is closely related to the *mcr-1.13* strain isolated from meat, and its spread could result in widespread resistance to colistin.

There are two limitations to this study: Firstly, the *E. coli* isolates were collected solely from fecal samples in Shanghai province, China. To obtain more accurate results, it would be beneficial to include isolates from additional regions with prolonged monitoring. Secondly, the fitness cost of ECPX221 needs to be assessed in order to evaluate the potential plasmid loss imposed by pECPX221.

## Conclusion

The study found that the prevalence of *mcr-1.1*-harboring *E. coli* among clinical fecal isolates in Shanghai is low. However, the strain ECPX221, which carries the *mcr-1.1* gene, exhibited extensive antimicrobial resistance profiles and additional resistance genes. The results of the conjugation experiment confirmed the horizontal transfer of the *mcr-1.1* gene, and the *mcr-1.1*-harboring plasmid pECPX221 was found to be stable in the recipient strain. Phylogenetic analysis showed an evolutionary linkage between MCR-1 and MCR-1 homolog proteins. Given the crucial role of colistin as a last-line treatment option against infections caused by multidrug-resistant Gram-negative bacteria, continuous surveillance is urgently needed to monitor the spread of the coexistence of the *mcr-1.1* gene and other important resistance genes.

## Data availability statement

The datasets presented in this study can be found in online repositories. The names of the repository/repositories and accession number(s) can be found at: https://www.ncbi.nlm.nih.gov/, PRJNA929103.

## Ethics statement

The studies involving humans were approved by Shanghai Municipal Center for Disease Control and Prevention. The studies were conducted in accordance with the local legislation and institutional requirements. Written informed consent for participation in this study was provided by the participants’ legal guardians/next of kin.

## Author contributions

JF wrote the draft, revised the manuscript, designed this study, and responsible for the whole experiment. HYW, YZ, JYL, YC, YTW, JYF, and QS participated in the whole experiment process. ZAY and MC managed the experiment and provided suggestions and revisions. All authors contributed to the article and approved the submitted version.

## Funding

The work was supported by the Three-Year Initiative Plan for Strengthening Public Health System Construction in Shanghai (2023-2025; Grant no. GWVI-3).

## Conflict of interest

The authors declare that the research was conducted in the absence of any commercial or financial relationships that could be construed as a potential conflict of interest.

## Publisher’s note

All claims expressed in this article are solely those of the authors and do not necessarily represent those of their affiliated organizations, or those of the publisher, the editors and the reviewers. Any product that may be evaluated in this article, or claim that may be made by its manufacturer, is not guaranteed or endorsed by the publisher.

## Footnotes

^1^ http://www.mgc.ac.cn/VFs/main.htm

^2^ https://card.mcmaster.ca/

^3^ https://cge.cbs.dtu.dk/services/PlasmidFinder/

^4^ https://pubmlst.org/

^5^ https://mjsull.github.io/Easyfig/

^6^ https://github.com/ParBLiSS/FastANI

^7^ http://www.clustal.org/
