# Supplementary material for: Stability and genetic insights of the co-existence of blaCTX-M-65, blaOXA-1, and mcr-1.1 harboring conjugative IncI2 plasmid isolated from a clinical extensively-drug resistant Escherichia coli ST744 in Shanghai
Source: Front Public Health. 2023 Aug 23;11:1216704. doi: 10.3389/fpubh.2023.1216704 (PMC10481164; doi:10.3389/fpubh.2023.1216704)
Supplement: Supplementary file 2 [file Table_2.DOCX]

>ECPX221

MMQHTSVWYRRSVSPFVLVASVAVFLTATANLTFFDKISQTYPIADNLGFVLTIAVVLFGAMLLITTLLSSYRYVLKPVLILLLIMGAVTSYFTDTYGTVYDTTMLQNALQTDQAETKDLLNAAFIMRIIGLGVLPSLLVAFVKVDYPTWGKGLMRRLGLIVASLALILLPVVAFSSHYASFFRVHKPLRSYVNPIMPIYSVGKLASIEYKKASAPKDTIYHAKDAVQATKPDMRKPRLVVFVVGETARADHVSFNGYERDTFPQLAKIDGVTNFSNVTSCGTSTAYSVPCMFSYLGADEYDVDTAKYQENVLDTLDRLGVSILWRDNNSDSKGVMDKLPKAQFADYKSATNNAICNTNPYNECRDVGMLVGLDDFVAANNGKDMLIMLHQMGNHGPAYFKRYDEKFAKFTPVCEGNELAKCEHQSLINAYDNALLATDDFIAQSIQWLQTHSNAYDVSMLYVSDHGESLGENGVYLHGMPNAFAPKEQRSVPAFFWTDKQTGITPMATDTVLTHDAITPTLLKLFDVTADKVKDRTAFIR

>WP_049589868.1 MCR-1.1 [Gammaproteobacteria]

MMQHTSVWYRRSVSPFVLVASVAVFLTATANLTFFDKISQTYPIADNLGFVLTIAVVLFGAMLLITTLLSSYRYVLKPVLILLLIMGAVTSYFTDTYGTVYDTTMLQNALQTDQAETKDLLNAAFIMRIIGLGVLPSLLVAFVKVDYPTWGKGLMRRLGLIVASLALILLPVVAFSSHYASFFRVHKPLRSYVNPIMPIYSVGKLASIEYKKASAPKDTIYHAKDAVQATKPDMRKPRLVVFVVGETARADHVSFNGYERDTFPQLAKIDGVTNFSNVTSCGTSTAYSVPCMFSYLGADEYDVDTAKYQENVLDTLDRLGVSILWRDNNSDSKGVMDKLPKAQFADYKSATNNAICNTNPYNECRDVGMLVGLDDFVAANNGKDMLIMLHQMGNHGPAYFKRYDEKFAKFTPVCEGNELAKCEHQSLINAYDNALLATDDFIAQSIQWLQTHSNAYDVSMLYVSDHGESLGENGVYLHGMPNAFAPKEQRSVPAFFWTDKQTGITPMATDTVLTHDAITPTLLKLFDVTADKVKDRTAFIR

>WP_129336087.1 MCR-1.19 [Salmonella enterica]

MMQHTSVWYRRSVSPFVLVASVAVFLTATANLTFFDKISQTYPIADNLGFVLTIAVVLFGAMLLITTLLSSYRYVLKPVLILLLIMGAVTSYFTDTYGTVYDTTMLQNALQTDQAETKDLLNAAFIMRIIGLGVLPSLLVAFVKVDYPTWGKGLMRRLGLIVASLALILLPVVAFSSHYASFFRVHKPLRSYVNPIMPIYSVGKLASIEYKKASAPKDTIYHAKDAVQATKPDMRKPRLVVFVVGETARADHVSFNGYERDTFPQLAKIDGVTNFSNVTSCGTSTAYSVPCMFSYLGADEYDVDTAKYQENVLDTLDRLGVSILWRDNNSDSKGVMDKLPKAQFADYKSATNNAICNTNPYNECRDVGMLVGLDDFVAANNGKDMLIMLHQMGNHGPAYFKRYDEKFAKFTPVCEGNELAKCEHQSLINAYDNALLATDDFIAQSIQWLQTHSNAYDVSMLYVSDHGESLGENGVYLHGMPNAFAPKEQRSVPAFFWTDKQTGITPMATDTILTHDAITPTLLKLFDVTADKVKDRTAFIR

>WP_220123500.1 MCR-1 [Klebsiella pneumoniae]

MMQHTSVWYRRSVSPFVLVASVAVFLTATANLTFFDKISQTYPIADNLGFVLTIAVVLFGAMLLITTLLSSYRYVLKPVLILLLIMGAVTSYFTDTYGTVYDTTMLQNALQTDQAETKDLLNAAFIMRIIGLGVLPSLLVAFVKVDYPTWGKGLMRRLGLIVASLALILLPVVAFSSHYASFFRVHKPLRSYVNPIMPIYSVGKLASIEYKKASAPKDTIYHAKDAVQATKPDMRKPRLVVFVVGETARADHVSFNGYERDTFPQLAKIDGVTNFSNVTSCGTSTAYSVPCMFSYLGADEYDVDTAKYQENVLDTLDRLGVSILWRDNNSDSKGVMDKLPKAQFADYKSTTNNAICNTNPYNECRDVGMLVGLDDFVAANNGKDMLIMLHQMGNHGPAYFKRYDEKFAKFTPVCEGNELAKCEHQSLINAYDNALLATDDFIAQSIQWLQTHSNAYDVSMLYVSDHGESLGENGVYLHGMPNAFAPKEQRSVPAFFWTDKQTGITPMATDTVLTHDAITPTLLKLFDVTADKVKDRTAFIR

>EAN4289655.1 MCR-1 [Salmonella enterica]

MMQHTSVWYRRSVSPFVLVASVAVFLTATANLTFFDKISQTYPIADNLGFVLTIAVVLFGAMLLITTLLSSYRYVLKPVLILLLIMGAVTSYFTDTYGTVYDTTMLQNALQTDQAETKDLLNAAFIMRIIGLGVLPSLLVAFVKVDYPTWGKGLMRRLGLIVASLALILLPVVAFSSHYASFFRVHKPLRSYVNPIMPIYSVGKLASIEYKKASAPKDTIYHAKDAVQATKPDMRKPRLVVFVVGETARADHVSFNGYERDTFPQLAKIDGVTNFSNVTSCGTSTAYSVPCMFSYLGADEYDVDTAKYQENVLDTLDRLGVSILWRDNNSDSKGVMDKLPKAQFADYKSATNNAICNTNPYNECRDVGMLVGLDDFVAANNGKDMLIMLHQMGNHGPAYFKRYDEKFAKFTPVCEGNELAKCEHQSLINAYDNALLATDDFIAQSIQWLQTHSNAYDVSMLYVSDHGESLGENGVYLHGMPNAFAPQEQRSVPAFFWTDKQTGITPMATDTVLTHDAITPTLLKLFDVTADKVKDRTAFIR

>WP_172694651.1 MCR-1 [Salmonella enterica]

MMQHTSVWYRRSVSPFVLVASVAVFLTATANLTFFDKISQTYPIADNLGFVLTIAVVLFGAMLLITTLLSSYRYVLKPVLILLLIMGAVTSYFTDTYGTVYDTTMLQNALQTDQAETKDLLNAAFIMRIIGLGVLPSLLVAFVKVDYPTWGKGLMRRLGLIVASLALILLPVVAFSSHYASFFRVHKPLRSYVNPIMPIYSVGKLASIEYKKASAPKDTIYHAKDAVQATKPDMRKPRLVVFVVGETARADHVSFNGYERDTFPQLAKIDGVTNFSNVTSCGTSTAYSVPCMFSYLGADEYDVDTAKYQENVLDTLDRLGVSILWRDNNSDSKGVMDKLPKAQFADYKSATNNAICNTNPYNECRDVGMLVGLDDFVAANNGKDMLIMLHQMGNHGPAYF

KRYDEKFAKFTPVCEGNELAKCEHQSLINAYDNALLATDDFIAQSIQWLQTHSNAYDVSMLYVSDHGESLGENGVYLHGMPNAFAPKEQRSVPAFFWTDKQTGITPVATDTVLTHDAITPTLLKLFDVTADKVKDRTAFIR

>HAE2238360.1 MCR-1 [Salmonella enterica subsp. enterica serovar Typhimurium]

MMQHTSVWYRRSVSPFVLVASVAVFLTATANLTFFDKISQTYPIADNLGFVLTIAVVLFGAMLLITTLLSSYRYVLKPVLILLLIMGAVTSYFTDTYGTVYDTTMLQNALQTDQAETKDLLNAAFIMRIIGLGVLPSLLVAFVKVDYPTWGKGLMRRLGLIVASLALILLPVVAFSSHYASFFRVHKPLRSYVNPIMPIYSVGKLASIEYKKASAPKDTIYHAKDAVQATKPDMRKPRLVVFVVGETARADHVSFNGYERDTFPQLAKIDGVTNFSNVTSCGTSTAYSVPCMFSYLGADEYDVDTAKYQENVLDTLDRLGVSILWRDNNSDSKGVMDKLPKAQFADYKSATNNAICNTNPYNECRDVGMLVGLDDFVAANNGKDMLIMLHQMGNHGPAYFKRYDEKFAKFTPVCEGNELAKCEHQSLINAYDNALLATDDFIAQSIQWLQMHSNAYDVSMLYVSDHGESLGENGVYLHGMPNAFAPKEQRSVPAFFWTDKQTGITPMATDTVLTHDAITPTLLKLFDVTADKVKDRTAFIR

>HBY7764053.1 MCR-1 [Klebsiella pneumoniae]

MMQHTSVWYRRSVSPFVLVASVAVFLTATANLTFFDKISQTYPIADNLGFVLTIAVVLFGAMLLITTLLSSYRYVLKPVLILLLIMGAVTSYFTDTYGTVYDTTMLQNALQTDQAETKDLLNAAFIMRIIGLGVLPSLLVAFVKVDYPTWGKGLMRRLGLIVASLALILLPVVAFSSHYASFFRVHKPLRSYVNPIMPIYSVGKLASIEYKKASAPKDTIYHAKDAVQATKPDMRKPRLVVFVVGETARADHVSFNGYERDTFPQLAKIDGVTNFSNVTSCGTSTAYSVPCMFSYLGADEYDVDTAKYQENVLDTLDRLGVSILWRDNNSDSKGVMDKLPKAQFADYKSATNNAICNTNPYNECRDVGMLVGLDDFVAANNGKDMLIILHQMGNHGPAYFKRYDEKFAKFTPVCEGNELAKCEHQSLINAYDNALLATDDFIAQSIQWLQTHSNAYDVSMLYVSDHGESLGENGVYLHGMPNAFAPKEQRSVPAFFWTDKQTGITPMATDTVLTHDAITPTLLKLFDVTADKVKDRTAFIR

>ECM8937208.1 MCR-1 [Salmonella enterica subsp. enterica serovar Typhimurium]

MMQHTSVWYRRSVSPFVLVARVAVFLTATANLTFFDKISQTYPIADNLGFVLTIAVVLFGAMLLITTLLSSYRYVLKPVLILLLIMGAVTSYFTDTYGTVYDTTMLQNALQTDQAETKDLLNAAFIMRIIGLGVLPSLLVAFVKVDYPTWGKGLMRRLGLIVASLALILLPVVAFSSHYASFFRVHKPLRSYVNPIMPIYSVGKLASIEYKKASAPKDTIYHAKDAVQATKPDMRKPRLVVFVVGETARADHVSFNGYERDTFPQLAKIDGVTNFSNVTSCGTSTAYSVPCMFSYLGADEYDVDTAKYQENVLDTLDRLGVSILWRDNNSDSKGVMDKLPKAQFADYKSATNNAICNTNPYNECRDVGMLVGLDDFVAANNGKDMLIMLHQMGNHGPAYFKRYDEKFAKFTPVCEGNELAKCEHQSLINAYDNALLATDDFIAQSIQWLQTHSNAYDVSMLYVSDHGESLGENGVYLHGMPNAFAPKEQRSVPAFFWTDKQTGITPMATDTVLTHDAITPTLLKLFDVTADKVKDRTAFIR

>HCJ4025449.1 MCR-1 [Salmonella enterica]

MMQHTSVWYRRSVSPFVLVASVAVFLTATANLTFFDKISQTYPIADNLGFVLTIAVVLFGAMLLITTLLSSYRYVLKPVLILLLIMGAVTSYFTDTYGTVYDTTMLQNALQTDQAETKDLLNAAFIMRIIGLGVLPSLLVAFVKVDYPTWGKGLMRRLGLIVASLALILLPVVAFSSHYASFFRVHKPLRSYVNPIMPIYSVGKLASIEYKKASAPKDTIYHAKDAVQATKPDMRKPRLVVFVVGETARADHVSFNGYERDTFPQLAKIDGVTNFSNVTSCGTSTAHSVPCMFSYLGADEYDVDTAKYQENVLDTLDRLGVSILWRDNNSDSKGVMDKLPKAQFADYKSATNNAICNTNPYNECRDVGMLVGLDDFVAANNGKDMLIMLHQMGNHGPAYFKRYDEKFAKFTPVCEGNELAKCEHQSLINAYDNALLATDDFIAQSIQWLQTHSNAYDVSMLYVSDHGESLGENGVYLHGMPNAFAPKEQRSVPAFFWTDKQTGITPMATDTVLTHDAITPTLLKLFDVTADKVKDRTAFIR

>HBW8909126.1 MCR-1 [Klebsiella pneumoniae]

MMQHTSVWYRRSVSPFVLVASVAVFLTATANLTFFDKISQTYPIADNLGFVLTIAVVLFGAMLLITTLLSSYRYVLKPVLILLLIMGAVTSYFTDTYGTVYDTTMLQNALQTDQAETKDLLNAAFIMRIIGLGVLPSLLVAFVKVDYPTWGKGLMRRLGLIVASLALILLPVVAFSSHYASFFRVHKPLRSYVNPIMPIYSVGKLASIEYKKASAPKDTIYHAKDAVQATKPDMRKPRLVVFVVGETARADHVSFNGYERDTFPQLAKIDGVTNFSNVTSCGTSTAYSVPCMFSYLGADEYDVDTAKYQENVLDTLDRLGVSILWRDNNSDSKGVIDKLPKAQFADYKSATNNAICNTNPYNECRDVGMLVGLDDFVAANNGKDMLIMLHQMGNHGPAYFKRYDEKFAKFTPVCEGNELAKCEHQSLINAYDNALLATDDFIAQSIQWLQTHSNAYDVSMLYVSDHGESLGENGVYLHGMPNAFAPKEQRSVPAFFWTDKQTGITPMATDTVLTHDAITPTLLKLFDVTADKVKDRTAFIR

>HAE2600850.1 MCR-1 [Salmonella enterica subsp. enterica serovar Typhimurium]

MMQHTSVWYRRSVSPFVLVASVAVFLTATANLTFFDKISQTYPIADNLGFVLTIAVVLFGAMLLITTLLSSYRYVLKPVLILLLIMGAVTSYFTDTYGTVYDTTMLQNALQTDQAETKDLLNAAFIMRIIGLGVLPSLLVAFVKVDYPTWGKGLMRRLGLIVASLALILLPVVAFSSHYASFFRVHKPLRSYVNPIMPIYSVGKLASIEYKKASAPKDTIYHAKDAVQATKPDMRKPRLVVFVVGETARADHVSFNGYERDTFPQLAKIDGVTNFSNVTSCGTSTAYSVPCMFSYLGADEYDVDTAKYQENVLDTLDRLGVSILWRDNNSDSKGVMDKLPKAQFADYKSATNNAICNTKPYNECRDVGMLVGLDDFVAANNGKDMLIMLHQMGNHGPAYFKRYDEKFAKFTPVCEGNELAKCEHQSLINAYDNALLATDDFIAQSIQWLQTHSNAYDVSMLYVSDHGESLGENGVYLHGMPNAFAPKEQRSVPAFFWTDKQTGITPMATDTVLTHDAITPTLLKLFDVTADKVKDRTAFIR

>WP_076611061.1 MCR-1.5 [Enterobacteriaceae]

MMQHTSVWYRRSVSPFVLVASVAVFLTATANLTFFDKISQTYPIADNLGFVLTIAVVLFGAMLLITTLLSSYRYVLKPVLILLLIMGAVTSYFTDTYGTVYDTTMLQNALQTDQAETKDLLNAAFIMRIIGLGVLPSLLVAFVKVDYPTWGKGLMRRLGLIVASLALILLPVVAFSSHYASFFRVHKPLRSYVNPIMPIYSVGKLASIEYKKASAPKDTIYHAKDAVQATKPDMRKPRLVVFVVGETARADHVSFNGYERDTFPQLAKIDGVTNFSNVTSCGTSTAYSVPCMFSYLGADEYDVDTAKYQENVLDTLDRLGVSILWRDNNSDSKGVMDKLPKAQFADYKSATNNAICNTNPYNECRDVGMLVGLDDFVAANNGKDMLIMLHQMGNHGPAYFKRYDEKFAKFTPVCEGNELAKCEHQSLINAYDNALLATDDFIAQSIQWLQTYSNAYDVSMLYVSDHGESLGENGVYLHGMPNAFAPKEQRSVPAFFWTDKQTGITPMATDTVLTHDAITPTLLKLFDVTADKVKDRTAFIR

>WP_109545052.1 MCR-1.14 [Klebsiella pneumoniae]

MMQHTSVWYRRSVSPFVLVASVAVFLTATANLTFFDKVSQTYPIADNLGFVLTIAVVLFGAMLLITTLLSSYRYVLKPVLILLLIMGAVTSYFTDTYGTVYDTTMLQNALQTDQAETKDLLNAAFIMRIIGLGVLPSLLVAFVKVDYPTWGKGLMRRLGLIVASLALILLPVVAFSSHYASFFRVHKPLRSYVNPIIPIYSVGKLASIEYKKASAPKDTIYHAKDAVQATKPDMRKPRLVVFVVGETARADHVSFNGYERDTFPQLAKIDGVTNFSNVTSCGTSTAYSVPCMFSYLGADEYDVDTAKYQENVLDTLDRLGVSILWRDNNSDSKGVMDKLPKAQFADYKSATNNAICNTNPYNECRDVGMLVGLDDFVAANNGKDMLIMLHQMGNHGPAYFKRYDEKFAKFTPVCEGNELAKCEHQSLINAYDNALLATDDFIAQSIQWLQTHSNAYDVSMLYVSDHGESLGENGVYLHGMPNAFAPKEQRSVPAFFWTDKQTGITPMATDTVLTHDAITPTLLKLFDVTADKVKDRTAFIR

>WP_077248208.1 MCR-1.6 [Salmonella enterica]

MMQHTSVWYRRSVSPFVLVASVAVFLTATANLTFFDKISQTYPIADNLGFVLTIAVVLFGAMLLITTLLSSYRYVLKPVLILLLIMGAVTSYFTDTYGTVYDTTMLQNALQTDQAETKDLLNAAFIMRIIGLGVLPSLLVAFVKVDYPTWGKGLMRRLGLIVASLALILLPVVAFSSHYASFFRVHKPLRSYVNPIMPIYSVGKLASIEYKKASAPKDTIYHAKDAVQATKPDMRKPRLVVFVVGETARADHVSFNGYERDTFPQLAKIDGVTNFSNVTSCGTSTAYSVPCMFSYLGADEYDVDTAKYQENVLDTLDRLGVSILWRDNNSDSKGVMDKLPKAQFADYKSATNNAICNTNPYNECRDVGMLVGLDDFVAANNGKDMLIMLHQMGNHGPAYFKRYDEKFAKFTPVCEGNELAKCEHQSLINAYDNALLATDDFIAQSIQWLQTHSNAYDVSMLYVSDHGESLGENGVYLHGMPNAFAPKEQRSVPAFFWTDKQTGITPMATDTVLTHDAITPTLLKLFDVTADKVKDHTAFIR

>WP_188331884.1 MCR-1.29 [uncultured bacterium]

MMQHTSVWYRRSVSPFVLVASVAVFLTATANLTFFDKISQTYPIADNLGFVLTIAVVLFGAMLLITTLLSSYRYVLKPVLILLLIMGAVTSYFTDTYGTVYDTTMLQNALQTDQAETKDLLNAAFIMRIIGLGVLPSLLVAFVKVDYPTWGKGLMRRLGLIVASLALILLPVVAFSSHYASFFRVHKPLRSYVNPIMPIYSVGKLASIEYKKASAPKDTIYHAKDAVQATKPDMRKPRLVVFVVGETARADHVSFNGYERDTFPQLAKIDGVTNFSNVTSCGTSTAYSVPCMFSYLGADEYDVDTAKYQENVLDTLDRLGVSILWRDNNSDSKGVMDKLPKAQFADYKSATNNAICNTNPYNECRDVGMLVGLDDFVAANNGKDMLIMLHQMGNHGSAYFKRYDEKFAKFTPVCEGNELAKCEHQSLINAYDNALLATDDFIAQSIQWLQTHSNAYDVSMLYVSDHGESLGENGVYLHGMPNAFAPKEQRSVPAFFWTDKQTGITPMATDTVLTHDAITPTLLKLFDVTADKVKDRTAFIR

>WP_188331885.1 MCR-1.30 [uncultured bacterium]

MMQHTSVWYRRSVSPFVLVASVAVFLTATANLTFFDKISQTYPIADNLGFVLTIAVVLFGAMLLITTLLSSYRYVLKPVLILLLIMGAVTSYFTDTYGTVYDTTMLQNALQTDQAETKDLLNAAFIMRIIGLGVLPSLLVAFVKVDYPTWGKGLMRRLGLIVASLALILLPVVAFSSHYASFFRVHKPLRSYVNPIMPIYSVGKLASIEYKKASAPKDTIYHAKDAVQATKPDMRKPRLVVFVVGETARADHVSFNGYERDTFPQLAKIDGVTNFSNVTSCGTSTAYSVPCMFSYLGADEYDVDTAKYQENVLDTLDRLGVSILWRDNNSDSKGVMDKLPKAQFADYKSATNNAICNTNPYNECRDVGMLVGLDDFVAANNGKDMLIMLHQMGNHGPAYFKRYDEKFAKFTPVCEGNELAKCEHQSLINAYDNALLATDDFIAQSIQWLQTHSNAYDVSMLYVSDHGESLGENDVYLHGMPNAFAPKEQRSVPAFFWTDKQTGITPMATDTVLTHDAITPTLLKLFDVTADKVKDRTAFIR

>WP_065274078.1 MCR-1.2 [Enterobacteriaceae]

MMLHTSVWYRRSVSPFVLVASVAVFLTATANLTFFDKISQTYPIADNLGFVLTIAVVLFGAMLLITTLLSSYRYVLKPVLILLLIMGAVTSYFTDTYGTVYDTTMLQNALQTDQAETKDLLNAAFIMRIIGLGVLPSLLVAFVKVDYPTWGKGLMRRLGLIVASLALILLPVVAFSSHYASFFRVHKPLRSYVNPIMPIYSVGKLASIEYKKASAPKDTIYHAKDAVQATKPDMRKPRLVVFVVGETARADHVSFNGYERDTFPQLAKIDGVTNFSNVTSCGTSTAYSVPCMFSYLGADEYDVDTAKYQENVLDTLDRLGVSILWRDNNSDSKGVMDKLPKAQFADYKSATNNAICNTNPYNECRDVGMLVGLDDFVAANNGKDMLIMLHQMGNHGPAYFKRYDEKFAKFTPVCEGNELAKCEHQSLINAYDNALLATDDFIAQSIQWLQTHSNAYDVSMLYVSDHGESLGENGVYLHGMPNAFAPKEQRSVPAFFWTDKQTGITPMATDTVLTHDAITPTLLKLFDVTADKVKDRTAFIR

>HAE6324312.1 MCR-1 [Salmonella enterica subsp. enterica serovar Typhimurium]

MMQHTSVWYRRSVSPFVLVASVAVFLTATANLTFFDKISQTYPIAVNLGFVLTIAVVLFGAMLLITTLLSSYRYVLKPVLILLLIMGAVTSYFTDTYGTVYDTTMLQNALQTDQAETKDLLNAAFIMRIIGLGVLPSLLVAFVKVDYPTWGKGLMRRLGLIVASLALILLPVVAFSSHYASFFRVHKPLRSYVNPIMPIYSVGKLASIEYKKASAPKDTIYHAKDAVQATKPDMRKPRLVVFVVGETARADHVSFNGYERDTFPQLAKIDGVTNFSNVTSCGTSTAYSVPCMFSYLGADEYDVDTAKYQENVLDTLDRLGVSILWRDNNSDSKGVMDKLPKAQFADYKSATNNAICNTNPYNECRDVGMLVGLDDFVAANNGKDMLIMLHQMGNHGPAYFKRYDEKFAKFTPVCEGNELAKCEHQSLINAYDNALLATDDFIAQSIQWLQTHSNAYDVSMLYVSDHGESLGENGVYLHGMPNAFAPKEQRSVPAFFWTDKQTGITPMATDTVLTHDAITPTLLKLFDVTADKVKDRTAFIR

>WP_213994615.1 MCR-1.32 [Enterobacteriaceae]

MVQHTSVWYRRSVGPFVLVASVAVFLTATANLTFFDKISQTYPIADNLGFVLTIAVVLFGAMLLITTLLSSYRYVLKPVLILLLIMGAVTSYFTDTYGTVYDTTMLQNALQTDQAETKDLLNAAFIMRIIGLGVLPSLLVAFVKVDYPTWGKGLMRRLGLIVASLALILLPVVAFSSHYASFFRVHKPLRSYVNPIMPIYSVGKLASIEYKKASAPKDTIYHAKDAVQATKPDMRKPRLVVFVVGETARADHVSFNGYERDTFPQLAKIDGVTNFSNVTSCGTSTAYSVPCMFSYLGADEYDVDTAKYQENVLDTLDRLGVSILWRDNNSDSKGVMDKLPKAQFADYKSATNNAICNTNPYNECRDVGMLVGLDDFVAANNGKDMLIMLHQMGNHGPAYFKRYDEKFAKFTPVCEGNELAKCEHQSLINAYDNALLATDDFIAQSIQWLQTHSNAYDVSMLYVSDHGESLGENGVYLHGMPNAFAPKEQRSVPAFFWTDKQTGITPMATDTVLTHDAITPTLLKLFDVTADKVKDRTAFIR

>WP_163397051.1 MCR-1.27 [Enterobacteriaceae]

MMQHTSVWCRRSVSPFVLVASVAVFLTATANLTFFDKISQTYPIADNLGFVLTIAVVLFGAMLLITTLLSSYRYVLKPVLILLLIMGAVTSYFTDTYGTVYDTTMLQNALQTDQAETKDLLNAAFIMRIIGLGVLPSLLVAFVKVDYPTWGKGLMRRLGLIVASLALILLPVVAFSSHYASFFRVHKPLRSYVNPIMPIYSVGKLASIEYKKASAPKDTIYHAKDAVQATKPDMRKPRLVVFVVGETARADHVSFNGYERDTFPQLAKIDGVTNFSNVTSCGTSTAYSVPCMFSYLGADEYDVDTAKYQENVLDTLDRLGVSILWRDNNSDSKGVMDKLPKAQFADYKSATNNAICNTNPYNECRDVGMLVGLDDFVAANNGKDMLIMLHQMGNHGPAYFKRYDEKFAKFTPVCEGNELAKCEHQSLINAYDNALLATDDFIAQSIQWLQTHSNAYDVSMLYVSDHGESLGENGVYLHGMPNAFAPKEQRSVPAFFWTDKQTGITPMATDTVLTHDAITPTLLKLFDVTADKVKDRTAFIR

>WP_116786830.1 MCR-1.15 [Klebsiella pneumoniae]

MQHTSVWYRRSVSPFVLVASVAVFLTATANLTFFDKISQTYPIADNLGFVLTIAVVLFGAMLLITTLLSSYRYVLKPVLILLLIMGAVTSYFTDTYGTVYDTTMLQNALQTDQAETKDLLNAAFIMRIIGLGVLPSLLVAFVKVDYPTWGKGLMRRLGLIVASLALILLPVVAFSSHYASFFRVHKPLRSYVNPIMPIYSVGKLASIEYKKASAPKDTIYHAKDAVQATKPDMRKPRLVVFVVGETARADHVSFNGYERDTFPQLAKIDGVTNFSNVKSCGTSTAYSVPCMFSYLGADEYDVDTAKYQENVLDTLDRLGVSILWRDNNSDSKGVMDKLPKAQFADYKSATNNAICNTNPYNECRDVGMLVGLDDFVAANNGKDMLIMLHQMGNHGPAYFKRYDEKFAKFTPVCEGNELAKCEHQSLINAYDNALLATDDFIAQSIQWLQTHSNAYDVSMLYVSDHGESLGENGVYLHGMPNAFAPKEQRSVPAFFWTDKQTGITPMATDTVLTHDAITPTLLKLFDVTADKVKDRTAFIR

>HAF7291450.1 MCR-1 [Salmonella enterica subsp. enterica serovar Typhimurium]

MMQHTSVWYRRSVSPFVLVASVAVFLTATANLTFFDKISQTYPIADNLGFVLTIAVVLFGAMLLITTLLSSYRYVLKPVLILLLIMGAVTSYFTDTYGTVYDTTMLQNALQTDQAETKDLLNAAFIMRIIGLGVLPSLLVAFVKVDYPTWGKGLMRRLGLIVASLALILLPVVAFSSHYASFFRVHKPLRSYVNPIMPIYSVGKLASIEYKKASAPKDTIYHAKDAVQATKPDMRKPRLVVFVVGETARADHVSFNGYERDTFPQLAKIDGVTNFSNVTSFGTSTAYSVPCMFSYLGADEYDVDTAKYQENVLDTLDRLGVSILWRDNNSDSKGVMDKLPKAQFADYKSATNNAICNTNPYNECRDVGMLVGLDDFVAANNGKDMLIMLHQMGNHGPAYFKRYDEKFAKFTPVCEGNELAKCEHQSLINAYDNALLATDDFIAQSIQWLQTHSNAYDVSMLYVSDHGESLGENGVYLHGMPNAFAPKEQRSVPAFFWTDKQTGITPMATDTVLTHDAITPTLLKLFDVTADKVKDRTAFIR

>WP_096807442.1 MCR-1.10 [Moraxella sp. MSG13-C03]

MVQHTSVWYRCSVSPFVLVASVSVFLTATANLTFFDKISQTYPIADNLGFVLTIAVVLFGAMLLITTLLSSYRYVLKPVLILLLIMGAVTSYFTDTYGTVYDTTMLQNALQTDQAETKDLLNAAFIMRIIGLGVLPSLLVAFVKVDYPTWGKGLVRRLGLIVASLALILLPVVAFSSHYASFFRVHKPLRSYVNPIMPIYSVGKLASIEYKKASAPKDTIYHAKDAVQATKPDTRKPRLVVFVVGETARADHVSFNGYERDTFPQLAKIDGVTNFSNVTSCGTSTAYSVPCMFSYLGADEYDVDTAKYQENVLDTLDRLGVSILWRDNNSDSKGVMDKLPKAQFADYKSATNNTICNTNPYNECRDVGMLVGLDDFVAANNGKDMLIMLHQMGNHGPAYFKRYDEKFAKFTPVCEGNELAKCEHQSLINAYDNALLATDDFITQSIQWLQTHSNAYDVSMLYVSDHGESLGENGVYLHGMPNAFAPKEQRSVPAFFWTDKQTGITPMATDTVLTHDAITPTLLKLFDVTADKVKDRTAFIR

>QIS31017.1 MCR-1.1 [uncultured bacterium]

MMQHTSVWYRRSVSPFVLVASVAVFLTATANLTFFDKISQTYPIADNLGFVLTIAVVLFGAMLLITTLLSSYRYVLKPVLILLLIMGAVTSYFTDTYGTVYDTTMLQNALQTDQAETKDLLNAAFIMRIIGLGVLPSLLVAFVKVDYPTWGKGLMRRLGLIVASLALILLPVVAFSSHYASFFRVHKPLRSYVNPIMPIYSVGKLASIEYKKASAPKDTIYHAKDAVQATKPDMRKPRLVVFVVGETARADHVSFNGYERDTFPQLAKIDGVTNFSNVTSCGTSTAYSVPCMFSYLGADEYDVDTAKYQENVLDTLDRLGVSILWRDNNSDSKGVMDKLPKAQFADYKSATNNAICNTNPYNECRDVGMLVGLDDFVAANNGKDMLKMGNHGPAYFKRYDEKFAKFTPVCEGNELAKCEHQSLINAYDNALLATDDFIAQSIQWLQTHSNAYDVSMLYVSDHGESLGENGVYLHGMPNAFAPKEQRSVPAFFWTDKQTGITPMATDTVLTHDAITPTLLKLFDVTADKVKDRTAFIR

>WP_160164897.1 MCR-1.23 [Salmonella enterica]

MMQHTSVWYRRSVIPFVLVASVAVFLTATANLTFFDKISQTYPIADNLGFVMTIAVLLFGPMLLITTMLSSHRYVLKTVLILLLIMDAVTNYFTDTYGTVYDTTMLQNALQTDQAETKDLLNAAFIMRIIGLGVLPSLLVAFVKVDYPTWGKGLMRRLGLIVASLALILLPVVAFSSHYASFFRVHKPLRSYVNPIMPIYSVGKLASIEYKKASAPKDTIYHAKDAVQATKPDMRKPRLVVFVVGETARADHVSFNGYERDTFPQLAKIDGVTNFSNVTSCGTSTAYSVPCMFSYLGADEYDVDTAKYQENVLDTLDRLGVSILWRDNNSDSKGVMDKLPKAQFADYKSATNNAICNTNPYNECRDVGMLVGLDDFVAANNGKDMLIMLHQMGNHGPAYFKRYDEKFAKFTPVCEGNELAKCEHQSLINAYDNALLATDDFIAQSIQWLQTHSNAYDVSMLYVSDHGESLGENGVYLHGMPNAFAPKEQRSVPAFFWTDKQTGITPMATDTVLTHDAITPTLLKLFDVTADKVKDRTAFIR
